# Synchronization in simplicial complexes of memristive Rulkov neurons

**DOI:** 10.3389/fncom.2023.1248976

**Published:** 2023-08-31

**Authors:** Mahtab Mehrabbeik, Sajad Jafari, Matjaž Perc

**Affiliations:** ^1^Department of Biomedical Engineering, Amirkabir University of Technology (Tehran Polytechnic), Tehran, Iran; ^2^Health Technology Research Institute, Amirkabir University of Technology (Tehran Polytechnic), Tehran, Iran; ^3^Faculty of Natural Sciences and Mathematics, University of Maribor, Maribor, Slovenia; ^4^Department of Medical Research, China Medical University Hospital, China Medical University, Taichung, Taiwan; ^5^Alma Mater Europaea, Maribor, Slovenia; ^6^Complexity Science Hub Vienna, Vienna, Austria; ^7^Department of Physics, Kyung Hee University, Seoul, Republic of Korea

**Keywords:** simplicial complex, higher-order network, memristive Rulkov, synchronization, cluster synchronization

## Abstract

Simplicial complexes are mathematical constructions that describe higher-order interactions within the interconnecting elements of a network. Such higher-order interactions become increasingly significant in neuronal networks since biological backgrounds and previous outcomes back them. In light of this, the current research explores a higher-order network of the memristive Rulkov model. To that end, the master stability functions are used to evaluate the synchronization of a network with pure pairwise hybrid (electrical and chemical) synapses alongside a network with two-node electrical and multi-node chemical connections. The findings provide good insight into the impact of incorporating higher-order interaction in a network. Compared to two-node chemical synapses, higher-order interactions adjust the synchronization patterns to lower multi-node chemical coupling parameter values. Furthermore, the effect of altering higher-order coupling parameter value on the dynamics of neurons in the synchronization state is researched. It is also shown how increasing network size can enhance synchronization by lowering the value of coupling parameters whereby synchronization occurs. Except for complete synchronization, cluster synchronization is detected for higher electrical coupling strength values wherein the neurons are out of the completed synchronization state.

## 1. Introduction

Understanding the complicated functions of the brain of an individual has been fascinating and challenging for scientists and academics (Changeux and Dehaene, 1989). One important component of this pursuit is the development of neural models capable of capturing the activity and functionality of individual neurons as well as the networks they compose (Aihara et al., 1990; Boccaletti et al., 2006; Majhi et al., 2019). Neuronal models provide a framework for investigating the brain's computational capacities, information processing, and cognitive function emergence (Ibarz et al., 2011). Over the years, several neuronal models have been proposed to describe and simulate the behavior of neurons, such as Hodgkin and Huxley (1952), FitzHugh (1961), Morris and Lecar (1981), and Hindmarsh and Rose (1984) models defined by differential equations and Chialvo (1995), Rulkov (2002), Izhikevich and Hoppensteadt (2004), and Zandi-Mehran et al. (2020) described by difference equations. The memristor has recently developed as a novel electrical component with enormous potential for neuronal modeling. In recent years, the memristor has emerged as a revolutionary electronic component with great potential for neuronal modeling (Lin et al., 2020; Ding et al., 2023; Xu et al., 2023). The potential of memristors to imitate crucial synaptic actions makes them significant for neuronal modeling. Synapses or connections amongst neurons, are critical in the brain's information analysis and learning process. They exhibit plasticity, indicating that their strength and efficiency can alter depending on the activity and timing of neuronal inputs. Neuronal models based on memristors, including memristive Hodgkin-Huxley (Hu and Liu, 2019), memristive FitzHugh-Nagumo (Chen et al., 2020), memristive Morris-Lecar (Bao et al., 2022), memristive Hindmarsh-Rose (Bao et al., 2018, 2021), memristive Rulkov (mRulkov) (Li et al., 2021), and memristive Chialvo (Vivekanandhan et al., 2023) neuron models, have enormous potential for various applications. They can, for instance, be used to study neurological disorders and replicate brain activity.

The study of complex systems has yielded essential insights into the structures and dynamics of numerous interconnected systems, ranging from social networks (Shahal et al., 2020) and biological systems (Ma and Tang, 2017) to network marketing and trading networks (Kim et al., 2006; Feng et al., 2014; Cho et al., 2023), in the discipline of network science. Networks have traditionally been depicted as graphs, with nodes and edges capturing entities and their pairwise connections (Burgio et al., 2020; Ghosh et al., 2022). Real-world systems, on the other hand, frequently demonstrate interactions and linkages that extend beyond simple pairwise connections (Ince et al., 2009; Alvarez-Rodriguez et al., 2021; Battiston et al., 2021). To capture the rich connectivity patterns in complex systems, researchers have turned to higher-order network representations (Majhi et al., 2022). Higher-order networks consider interactions among groups of nodes rather than just pairwise connections, enabling a more comprehensive understanding of complex systems (Carletti et al., 2020; Lotito et al., 2022). One powerful mathematical framework for modeling higher-order networks is simplicial complexes (Skardal and Arenas, 2020; Gambuzza et al., 2021). A simplicial complex is a mathematical structure that reflects the interactions between nodes at various granularities (Gambuzza et al., 2021). It expands the graph concept by integrating higher-order interactions such as triangles, tetrahedra, and higher-dimensional simplices (Ghorbanchian et al., 2021). Each simplex in a simplicial complex represents a group of nodes connected in a particular way. A triangle, for example, depicts a group of three nodes, each connected to the other two. By leveraging simplicial complexes, researchers can analyze and characterize complex systems more nuancedly. Higher-order networks derived from simplicial complexes allow for exploring intricate relationships that might not be evident when considering only pairwise connections (Battiston et al., 2021). These higher-order structures provide a more detailed description of the system's organization and dynamics, leading to a deeper understanding of complex systems.

Synchronization is a phenomenon where elements in a system coordinate their behavior to achieve coherence (Boccaletti et al., 2002). This fundamental phenomenon can be witnessed in a variety of complex systems, including biological (Li et al., 2022), social (Sorrentino and Ott, 2007), and technological (Sivrikaya and Yener, 2004) networks. While much study has concentrated on the synchronization in conventional pairwise interactions (Lin et al., 2021; Fan et al., 2022, 2023), there is rising interest in understanding synchronization in higher-order networks that capture interactions beyond pairwise links (Battiston et al., 2020; Bick et al., 2023; Boccaletti et al., 2023). The goal of studying synchronization in higher-order networks is to decipher the intricate dynamics, emergent behaviors, and collective phenomena resulting from higher-order interactions. This emerging subject studies synchronization's emergence, development, and evolution in complex network structures such as simplicial complexes and hypergraphs. Researchers have been investigating synchronization in higher-order networks to unravel the fundamental principles driving collective dynamics and information processing in various fields spanning from neuroscience to social networks and beyond. For instance, Skardal et al. (2021) addressed how higher-order interactions effectively help to achieve optimized synchronization. Employing a random network of 500 phase oscillators optimized in order to get strongly synchronized, they found that strengthening the higher-order interactions resulted in improving the optimal synchronization. On the other hand, according to Gallo et al. (2022), directed higher-order interactions were reported as an impediment to attaining synchrony; otherwise, such interactions may stabilize unstable synchronized states. More clearly, studying a higher-order network of 8 Rössler oscillators structures in a random graph, the authors noticed that applying directed higher-order interactions could ruin the synchronization state. In contrast, as the asymmetry varied, synchronization was achievable. Multiplex higher-order network was the subject that Anwar and Ghosh (2022) pursued. They looked for synchronization in a two-layer network within each layer, the 500 Rössler oscillators interacting via diffusive pairwise and non-pairwise connections and structured in a scale-free configuration. Their results portrayed that higher-order interactions enhance intra-layer synchronization, and inter-layer synchronization becomes more robust compared to the pure pairwise case. Focusing on higher-order neuronal networks, Parastesh et al. (2022) analyzed the synchronization of a fully connected network of 20 Hindmarsh-Rose neurons interacting through two- and three-node interactions. Assuming different non-pairwise (electrical and chemical) interactions in combination with diffusive (electrical) pairwise connections, they showed that even weak second-order interactions could facilitate synchronization by reducing the pairwise strengths needed for achieving synchrony. In this regard, Mirzaei et al. (2022) pictured that scaling the synchronization patterns to lower coupling strength values was feasible via higher-dimensional interactions. To demonstrate the impact of adding three-, four-, and five-node chemical interactions stepwise on the synchronization state, they took into account a fully connected network of five conventional Rulkov maps with two-node diffusive inner linking and chemical connections. Mehrabbeik et al. (2023) recently investigated the synchronization of different higher-order networks made up of 10 Hindmarsh-Rose maps. The authors intended to figure out the impact of higher-order synaptic functions applied to two-node and three-node communication on network global synchronization. As a result, it was discovered that chemical synapses greatly improved synchronization compared to diffusively defined synapses, which include electrical and inner-linking functions.

Through the use of electrical and chemical synapses on two- and three-node interactions, the present study addresses synchronization in a higher-order network of mRulkov maps. The acquired results are compared to the case where electrical and chemical synapses are devoted simultaneously to two-node connections called hybrid synapses. The consequence of increasing the network size on synchronization state and synchronization manifold dynamics is additionally considered. Furthermore, the largest network (50 nodes) is sought for other synchronization patterns. The following is how the paper is set up: The mRulkov model and its dynamics are fully clarified in Section 2. In Sections 3 and 4, the subjected higher-order network model's linear stability analysis is covered in detail. Results are presented in Section 5, and Section 6 recaps the paper by emphasizing the findings.

## 2. The mRulkov neuron map

The mRulkov map is a mathematical neuron model that combines the dynamics of the 2D Rulkov map with the memristive nonlinearity proposed by Li et al. (2021). The original Rulkov map is a discrete-time dynamical system that exhibits complicated behavior, such as chaos, and is frequently used to resemble biological systems. The mRulkov model is a piecewise nonlinear model described by three dependent nonlinear difference equations as follows:


(1) $$\begin{array}{l} \{\begin{array}{l} x(n+1)=\\ \{\begin{array}{ll} \frac{\alpha }{1-x(n)}+y(n) & x(n)\leq 0\\ \alpha +y(n) & 0<x(n)<\alpha +y(n)\\ -1 & x(n)\geq \alpha +y(n) \end{array},\\ y(n+1)=y(n)-\beta (x(n)-\rho +1), \end{array} \end{array}$$

where *x*(*n*) and *y*(*n*) are the membrane potential and recovery state variables at time step *n*, and α, and β are parameters that determine the system's behavior. Also, the parameter ρ involves the effect of external factors on the model.

**Figure 1:**
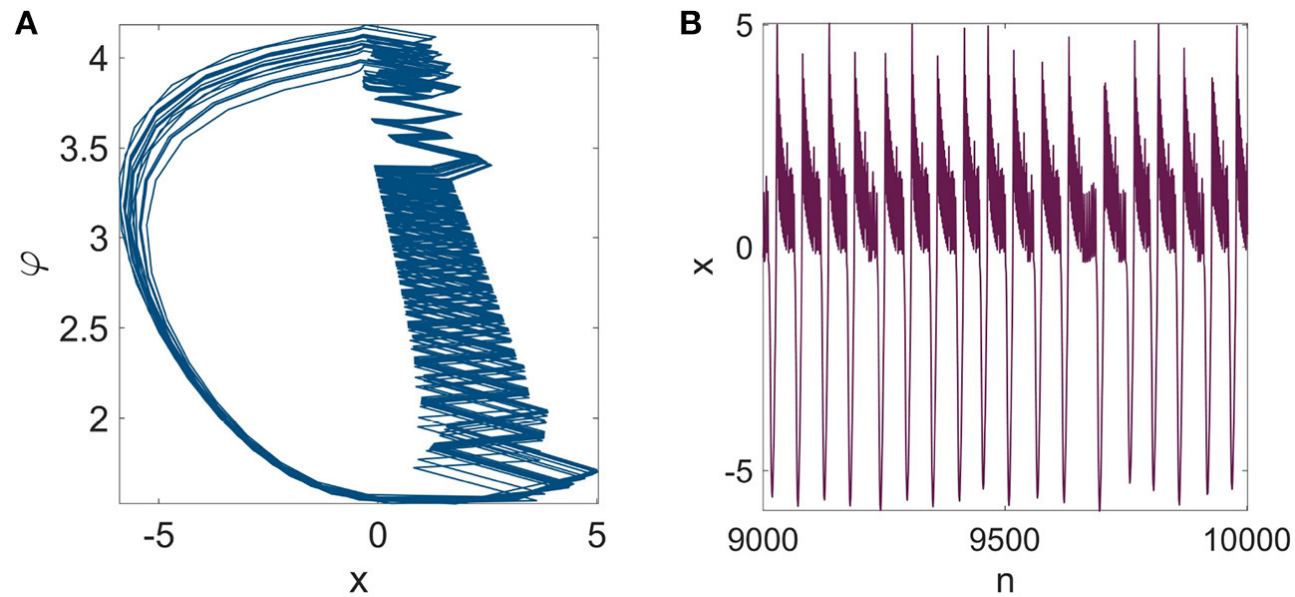
The dynamical behavior of the mRulkov neuron model represented in **(A)** *x*-*φ* state space and **(B)** time series of variable *x* over 10, 000 iterations. The parameters are set at [α, β, ε, *μ*] = (5, 0.05, 0.05, 0.55) and the initial condition is [*x*(1), *y*(1), *φ*(1)] = (0, 0, 0). In the circumstances that were taken into consideration, the mRulkov model exhibited chaotic behavior. When the mRulkov model is operating in bursting mode, it is possible to witness this chaotic dynamic.

The addition of memristive nonlinearity to the Rulkov map results in a system that exhibits even more complex behavior, including hyperchaotic behavior and the emergence of extreme multistability (Li et al., 2021). The mathematical expression of the mRulkov map proposed in Li et al. (2021), to which the flux-controlled memristor is applied, is given as follows:


(2) $$\begin{array}{l} \{\begin{array}{l} x(n+1)=f(x(n),y(n),\varphi (n))=\\ \mu \tanh(\phi (n))x(n)+\\ \{\begin{array}{ll} \frac{\alpha }{1-x(n)}+y(n) & x(n)\leq 0\\ \alpha +y(n) & 0<x(n)<\alpha +y(n)\\ -1 & x(n)\geq \alpha +y(n) \end{array},\\ y(n+1)=g(x(n),y(n),\varphi (n))=\\ y(n)-\beta x(n),\\ \varphi (n+1)=h(x(n),y(n),\varphi (n))=\\ \varphi (n)+\varepsilon x(n), \end{array} \end{array}$$

where *φ* is the flux variable, and *μ* is the magnetic induction strength induced by the membrane potential. For simplicity, ρ = 0 is considered in System (2). Considering α = 5, β = ε = 0.05, and *μ* = 0.55, the Rulkov model exhibits a chaotic dynamic as represented in Figure 1.

## 3. Discrete network model

**Figure 2:**
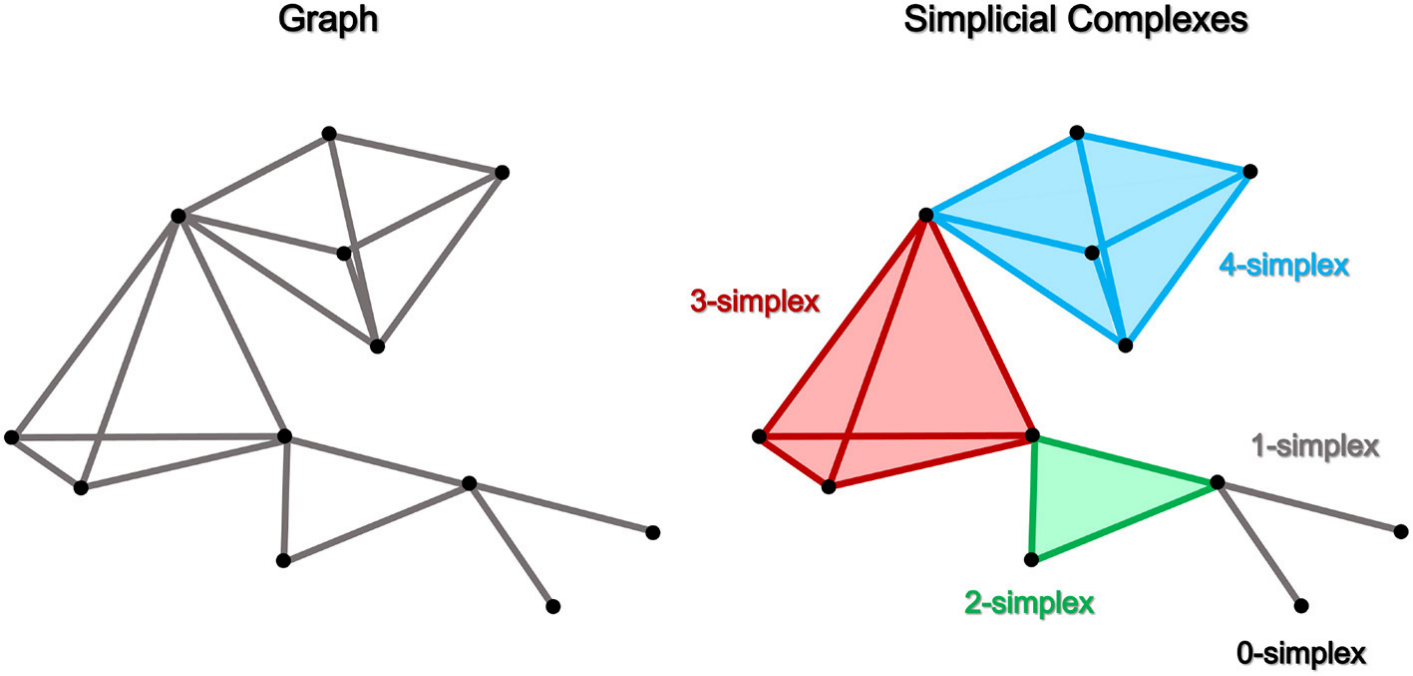
A schematic representation of a traditional network with only pairwise interactions defined by edges and links and a network with higher-order (non-pairwise) interactions defined by simplicial complexes. As a result, a *d*-simplex can represent a group of *d*+1 nodes interacting in a specific way. In higher-order network structures, nodes/vertices are 0-simplex (shown in black), links are 1-simplex (shown in gray), triangles are 2-simplex (shown in green), tetrahedra are 3-simplex (shown in red), pentahedra are 4-simplex (shown in blue), and polyhedra are d-simplex. In this example, it is possible to observe how simplicial complexes can be derived in a conventional graph-based network design, and the contrasts between the two approaches are underlined.

Simplicial complexes, geometric objects that generalize triangles and tetrahedra to higher dimensions, are a powerful tool for representing higher-order interactions between groups of elements in a network. In a simplicial complex, each element is represented by a vertex, and higher-order interactions are represented by higher-dimensional simplices, such as edges (1-simplex), triangles (2-simplex), tetrahedra (3-simplex), pentahedra (4-simplex), and other polyhedra. As indicated by Figure 2, simplicial complexes allow for the representation of more complex relationships between elements than is possible with traditional network representations. Only links color-coded in gray have the key role in defining the structure of a network, as seen in Figure 2 (left panel). On the other hand, as shown in the right panel, a *d*-dimensional simplicial complex is formed when a set of nodes join together to form a morphological object with *d* faces. These linked neurons can also establish a novel network topology in which they interact in novel ways. For instance, a 2-simplex structure is formed when three nodes are joined together to create a triangle, and interactions involving three nodes must be taken into account.

The mathematical definition of a *d*-dimensional simplicial complex in the discrete domain is expressed as:


(3) $$\begin{array}{l} X_{i}(n+1)=F(X_{i}(n))+\\ \sigma_{1}\sum_{j_{1}=1}^{N}A_{ij_{1}}^{\left(1\right)}G^{\left(1\right)}(X_{i}(n),X_{j_{1}}(n))+\\ \sigma_{2}\sum_{j_{1}=1}^{N}\sum_{j_{2}=1}^{N}A_{ij_{1}j_{2}}^{\left(2\right)}G^{\left(2\right)}(X_{i}(n),X_{j_{1}}(n),X_{j_{2}}(n))+...+\\ \sigma_{d}\sum_{j_{1}=1}^{N}...\sum_{j_{d}=1}^{N}A_{ij_{1}...j_{d}}^{\left(d\right)}G^{\left(d\right)}(X_{i}(n),X_{j_{1}}(n),...,X_{j_{d}}(n)). \end{array}$$

Here, ${\text{X}}_{i}\left(n\right)={\left[x_{i}\left(n\right),y_{i}\left(n\right),\varphi_{i}\left(n\right)\right]}^{T}$, where *X*∈*R*^3^, contains the system's state variables belonging to the *i*-the system, *F*[*X*_*i*_(*n*)] =[*f*(*X*_*i*_(*n*)), *g*(*X*_*i*_(*n*)), *h*(*X*_*i*_(*n*))], where *F*:*R*^3^→*R*^3^, determines the nodes' dynamics, *N* is the number of nodes, and σ_1_, ..., σ_*d*_ are the coupling strength of interactions defined by 1 to *d* dimensional simplices. The network structure is defined by adjacency tensors *A*^(1)^, ..., *A*^(*d*)^, where $A^{\left(d\right)}=\left[A_{ij_{1}...j_{d}}^{\left(d\right)}\right]$ (Wei and Ding, 2016; Gambuzza et al., 2021). Adjacency tensors are a generalization of adjacency matrices to higher-order networks. In an adjacency matrix, each element represents the presence or absence of a connection between a pair of nodes in a network, while in an adjacency tensor, each element represents the presence or absence of a connection between a set of nodes of arbitrary size (Lucas et al., 2020). For example, $A_{ij_{1}}^{\left(1\right)}=1$ indicates that the *i*-th and *j*_1_-th nodes are connected through a link while $A_{ij_{1}...j_{d}}^{\left(d\right)}=1$ shows that nodes with indices *i, j*_1_, ..., *j*_*d*_ combine to form a polyhedron (*d*-simplex; Mirzaei et al., 2022; Parastesh et al., 2022; Mehrabbeik et al., 2023). Furthermore, *G*^(1)^, ..., *G*^(*d*)^ are the coupling functions describing the type of interactions among 2, ..., *d*+1 units building simplicial complexes.

A traditional network of *N* mRulkov maps coupled via hybrid (electrical and chemical) pairwise interactions $\left(G_{1}^{\left(1\right)}(X_{i}(n),X_{j}(n))=[x_{j}(n)-x_{i}(n),0,0]T\right]$ and $G_{2}^{\left(1\right)}\left(X_{i}(n),X_{j}(n)\right)=\left[(v-x_{i}(n))\Gamma (x_{j}(n)),0,0\right])$ in a global configuration is mathematically depicted as follows:


(4) $$\{\begin{array}{l} x_{i}(n+1)=f(X_{i}(n))+\\ \sigma_{1}\sum_{j=1}^{N}A_{ij}^{\left(1\right)}(x_{j}(n)-x_{i}(n))+\\ \sigma_{2}(v-x_{i}(n))\sum_{j=1}^{N}A_{ij}^{\left(1\right)}\Gamma (x_{j}(n)),\\ y_{i}(n+1)=g(X_{i}(n)),\\ \varphi_{i}(n+1)=h(X_{i}(n)), \end{array}$$

where *v* is the reversal potential, $\Gamma \left(x\right)=\frac{1}{1+e^{-r\left(x-\theta \right)}}$ is the chemical synaptic function that describes the kinetics of the neurotransmitter release and modulates the synaptic transmission using a sigmoidal function with a slope of *r* and a firing threshold of θ. The expression [*v*−*x*_*i*_(*n*)]Γ[*x*_*j*_(*n*)] represents the postsynaptic response at a synapse, where *x*_*i*_(*n*) and *x*_*j*_(*n*) are, respectively, the postsynaptic and presynaptic membrane potentials. The reversal potential *v* represents the neurotransmitter's equilibrium potential at the synapse. There is no net flow of ions when *v* = *x*_*i*_(*n*), and the synapse is said to be at its reversal potential. Moreover, Γ[*x*_*j*_(*n*)] denotes synaptic conductance and is influenced by several variables, including presynaptic activity. It influences how strongly neurotransmitters bind to receptors, affecting synaptic transmission and the postsynaptic membrane potential (Yamakou et al., 2020). If *v*−*x*_*i*_(*n*)>0, it results in depolarization (excitatory response), while if *v*−*x*_*i*_(*n*) < 0, it leads to hyperpolarization (inhibitory response; Shafiei et al., 2020). This response determines whether an action potential is generated a nd influences information transmission and processing within the neuronal network. Nonetheless, based on the general definition in Equation (3), considering a higher-order network of *N* globally coupled mRulkov neuron maps with first-order electrical connections $\left({\text{G}}^{\left(1\right)}\left({\text{X}}_{i}\left(n\right),{\text{X}}_{j}\left(n\right)\right)={\left[x_{j}\left(n\right)-x_{i}\left(n\right),0,0\right]}^{T}\right)$ and second-order chemical interactions (${\text{G}}^{\left(2\right)}\left({\text{X}}_{i}\left(n\right),{\text{X}}_{j}\left(n\right),{\text{X}}_{k}\left(n\right)\right)=\left[\left(v-x_{i}\left(n\right)\right)\left(\Gamma \left(x_{j}\left(n\right)\right)\Gamma \left(x_{k}\left(n\right)\right)\right),0,0\right]$), Network (4) changes into the following equations:


(5) $$\begin{array}{l} \{\begin{array}{l} x_{i}(n+1)=f(X_{i}(n))+\\ \sigma_{1}\sum_{j=1}^{N}A_{ij}^{\left(1\right)}(x_{j}(n)-x_{i}(n))+\sigma_{2}(v-x_{i}(n))\times \\ \sum_{j=1}^{N}\sum_{k=1}^{N}A_{ijk}^{\left(2\right)}(\Gamma (x_{j}(n))\Gamma (x_{k}(n))),\\ y_{i}(n+1)=g(X_{i}(n)),\\ \varphi_{i}(n+1)=h(X_{i}(n)). \end{array} \end{array}$$

In Networks (4) and (5), σ_1_ and σ_2_ denote the strength of the electrical and chemical synapses, respectively. Here, *x*_*j*_(*n*) and *x*_*k*_(*n*) are both considered as the postsynaptic neurons responsible for the release of the same neurotransmitters into the synaptic cleft of the postsynaptic neuron *x*_*i*_(*n*). For the subsequent investigations, the chemical synaptic parameters are set at *v* = θ = −1.4 and *r* = 50. The present study focuses on the collective dynamics of Network (5) to find the impact of higher-order interactions.

## 4. Stability analysis

The master stability function (MSF) is considered to assess the neurons' stability in the synchronization state (Pecora and Carroll, 1998). The MSF, which is influenced by the topology of the network, the degree of coupling between nodes, and the dynamics of each node, characterizes the linear stability of a synchronized state in a network. This function provides the necessary criteria for network synchronization by linearizing the dynamics of the network around the synchronized state and analyzing the resulting eigenvalues of the linearized system (Pecora and Carroll, 1998). To find the linearized system, a negligible perturbation δ*X*(*n*) = [δ*x*(*n*), δ*y*(*n*), δ*φ*(*n*)]^*T*^ is locally added to the neurons in their synchronous state **X**^*s*^(*n*) = [*x*^*s*^(*n*), *y*^*s*^(*n*), *φ*^*s*^(*n*)]*T*. Thus, $\delta {\text{X}}_{i}\left(n\right)={\text{X}}_{i}\left(n\right)-{\text{X}}^{s}\left(n\right)$. Assuming V as a matrix with columns that are the eigenvectors of the Laplacian matrix generated from the adjacency matrix, the transformation $\eta \left(n\right)=V^{-1}\delta {\text{X}}_{i}\left(n\right)$ leads to the desired linearized system.

In the first case, wherein no non-pairwise interactions are involved, and neurons are interacting through pairwise hybrid synapses (Network 4), the linearized system becomes:


(6) $$\begin{array}{l} \{\begin{array}{l} \eta^{x}(n+1)=Jf(X^{s}(n))-\sigma_{1}N\eta^{x}(n)-\\ \sigma_{2}((v-x^{s}(n))\Gamma_{x}(x^{s}(n))+\\ (N-1)\Gamma (x^{s}(n)))\eta^{x}(n),\\ \eta^{y}(n+1)=Jg(X^{s}(n)),\\ \eta^{\varphi }(n+1)=Jh(X^{s}(n)). \end{array} \end{array}$$

where *JF*[*X*^*s*^(*n*)] is the 3 × 3 Jacobian matrix of *F*[*X*(*n*)] evaluated at *X*^*s*^(*n*) and $\Gamma_{x}\left[x^{s}\left(n\right)\right]$ is the derivative of Γ(*x*) in the synchronization state *x*^*s*^(*n*). When all neurons evolve synchronously, ${\text{X}}_{1}\left(n\right)={\text{X}}_{2}\left(n\right)=...={\text{X}}_{N}\left(n\right)={\text{X}}^{s}\left(n\right)$, and thus, ${\text{G}}_{1}^{\left(1\right)}\left[{\text{X}}^{s}\left(n\right),{\text{X}}^{s}\left(n\right)\right]\equiv 0$ and ${\text{G}}_{2}^{\left(1\right)}\left[{\text{X}}^{s}\left(n\right),{\text{X}}^{s}\left(n\right)\right]\equiv \left[\left(v-x^{s}\left(n\right)\right)\Gamma \left(x^{s}\left(n\right)\right),0,0\right]$. Also, for a global network of *N* nodes, we have $\overset{N}{\underset{j=1}{\sum }}A_{ij}^{\left(1\right)}=N-1$. Therefore, the dynamics of the synchronous neurons with hybrid synapses obey the following system:


(7) $$\{\begin{array}{l} x^{s}(n+1)=Jf(X^{s}(n))+\\ \sigma_{2}(N-1)(v-x^{s}(n))\Gamma (x^{s}(n)),\\ y^{s}(n+1)=Jg(X^{s}(n)),\\ \varphi^{s}(n+1)=Jh(X^{s}(n)), \end{array}$$

which differs from the dynamics of an individual mRulkov neuron.

In the second case, wherein the non-pairwise chemical interactions, as well as the pairwise electrical connections, are involved (Network 5), the linearized system change into:


(8) $$\begin{array}{l} \{\begin{array}{l} \eta^{x}(n+1)=Jf(X^{s}(n))-\sigma_{1}N\eta^{x}(n)-\\ \sigma_{2}(N-2)(2(v-x^{s}(n))\times \\ (\Gamma_{x}(x^{s}(n)\Gamma (x^{s}(n)))+\\ (N-1)\Gamma^{2}(x^{s}(n)))\eta^{x}(n),\\ \eta^{y}(n+1)=Jg(X^{s}(n)),\\ \eta^{\varphi }(n+1)=Jh(X^{s}(n)), \end{array} \end{array}$$

In the synchronization state, since ${\text{X}}_{1}\left(n\right)={\text{X}}_{2}\left(n\right)=...={\text{X}}_{N}\left(n\right)={\text{X}}^{s}\left(n\right)$, *G*^(1)^[*X*^*s*^(*n*), *X*^*s*^(*n*)]≡0 and *G*^(2)^[*X*^*s*^(*n*), *X*^*s*^(*n*), *X*^*s*^(*n*)]≡[(*v*−*x*^*s*^(*n*))Γ^2^(*x*^*s*^(*n*)), 0, 0]. Moreover, due to the global coupling scheme, we have $\overset{N}{\underset{j=1}{\sum }}\overset{N}{\underset{k=1}{\sum }}A_{ijk}^{\left(2\right)}=\left(N-1\right)\left(N-2\right)$. Thus, the neurons' dynamics in the synchronization state can be described as follows:


(9) $$\begin{array}{l} \{\begin{array}{l} x^{s}(n+1)=Jf(X^{s}(n))+\\ \sigma_{2}(N-1)(N-2)(v-x^{s}(n))\Gamma (x^{s}(n)),\\ y^{s}(n+1)=Jg(X^{s}(n)),\\ \varphi^{s}(n+1)=Jh(X^{s}(n)), \end{array} \end{array}$$

which is different from System (7) or even an isolated neuron behavior.

The negative maximum Lyapunov exponent (Λ) of the linearized systems described in Systems (6) and (8) shows the stability of the synchronization state since the locally injected perturbations achieve zero, which means that the neurons remain in their synchronous state. The Appendix provides more information on how to conduct MSF analysis for both traditional and higher-order networks.

## 5. Results

Using the MSF analysis detailed in Section 4, here, the effect of applying higher-order interaction is investigated by examining Networks (4) and (5). The averaged synchronization error is also numerically determined in addition to the MSF technique, which analytically provides the essential synchronization criteria for the assessed networks to confirm the outcomes of the MSF method. The following definition applies to the averaged synchronization error:


(10) $$E={\langle \frac{1}{N-1}\sum_{j=2}^{N}\|X_{j}(n)-X_{1}(n)\|\rangle }_{n},$$

where 〈...〉 and ||...|| are the functions calculating the averaged value over discrete time steps *n* and the Euclidean norm, respectively.

**Figure 3:**
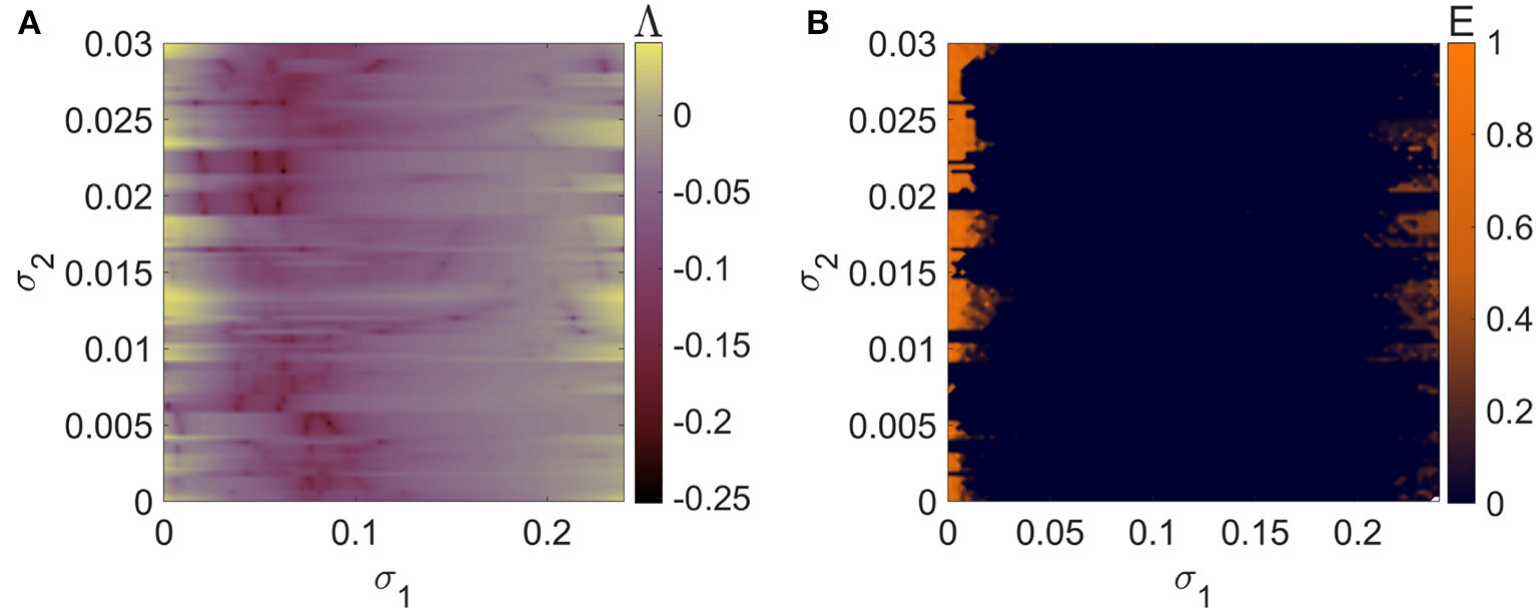
The criteria for synchronizing a network of *N* = 5 globally coupled mRulkov neuron maps with pairwise hybrid synapses represented by 2D **(A)** maximum Lyapunov exponent of the linearized System (6) and **(B)** averaged synchronization error of Network (4) in σ_1_-σ_2_ parameter plane. Although there is a significant amount of the examined parameter plane that is occupied by a synchronous region, the neurons break out of total asynchrony for values of σ_1_ that are either very small or very high in value. Thus, the value of σ_1_ playing a key role in synchronization incidence.

**Figure 4:**
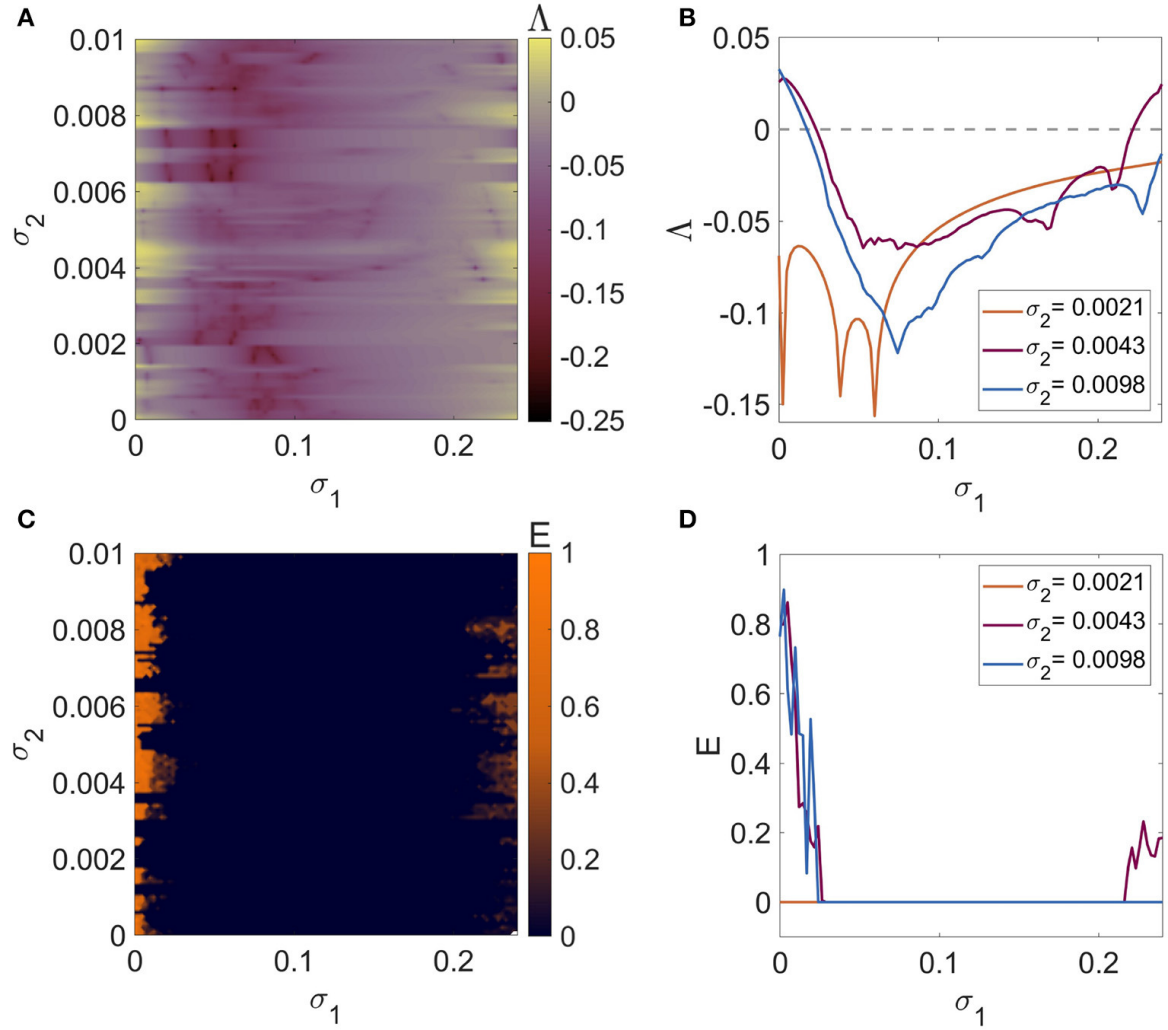
The criteria for synchronizing a network of *N* = 5 globally coupled mRulkov neuron maps with pairwise electrical and non-pairwise chemical synapses represented by 1D and 2D **(A, B)** maximum Lyapunov exponent of the linearized System (8) and **(C, D)** averaged synchronization error of Network (5). The 2D representations are in the parameter plane σ_1_-σ_2_ and the 1D curves are according to σ_1_ for σ_2_ = 0.0021, 0.0043, and 0.0098. The synchronization patterns are scaled to lower values of σ_2_ as the higher-order chemical interactions are added to the pairwise electrical connections. As a result, synchronization is improved as a result of its incidence being detected in second-order interactions with weaker strengths.

Figure 2 depicts the regions in the parameter plane σ_1_-σ_2_ (0 ≤ σ_1_ ≤ 0.24 and 0 ≤ σ_2_ ≤ 0.03) wherein *N* = 5 mRulkov neurons interacting through the hybrid synapses [Network (4)] are completely synchronous. Such region can be detected for Λ < 0 (Figure 3A) and *E* = 0 (Figure 3B). Similarly, Figure 4 provides the results of Lyapunov analysis of System (8) and synchronization error calculation of Network (5) in which the chemical synapse are considered as the second-order interactions alongside the first-order electrical connections for 0 ≤ σ_1_ ≤ 0.24 and 0 ≤ σ_2_ ≤ 0.01. Figure 4 reveals that when second-order chemical interactions are involved with the pairwise electrical connections, weaker strength of chemical couplings is needed to have the same synchronization patterns as shown in Figure 3. More clearly, higher-order interactions enhance synchronization by scaling the patterns to lower values of σ_2_.

**Figure 5:**
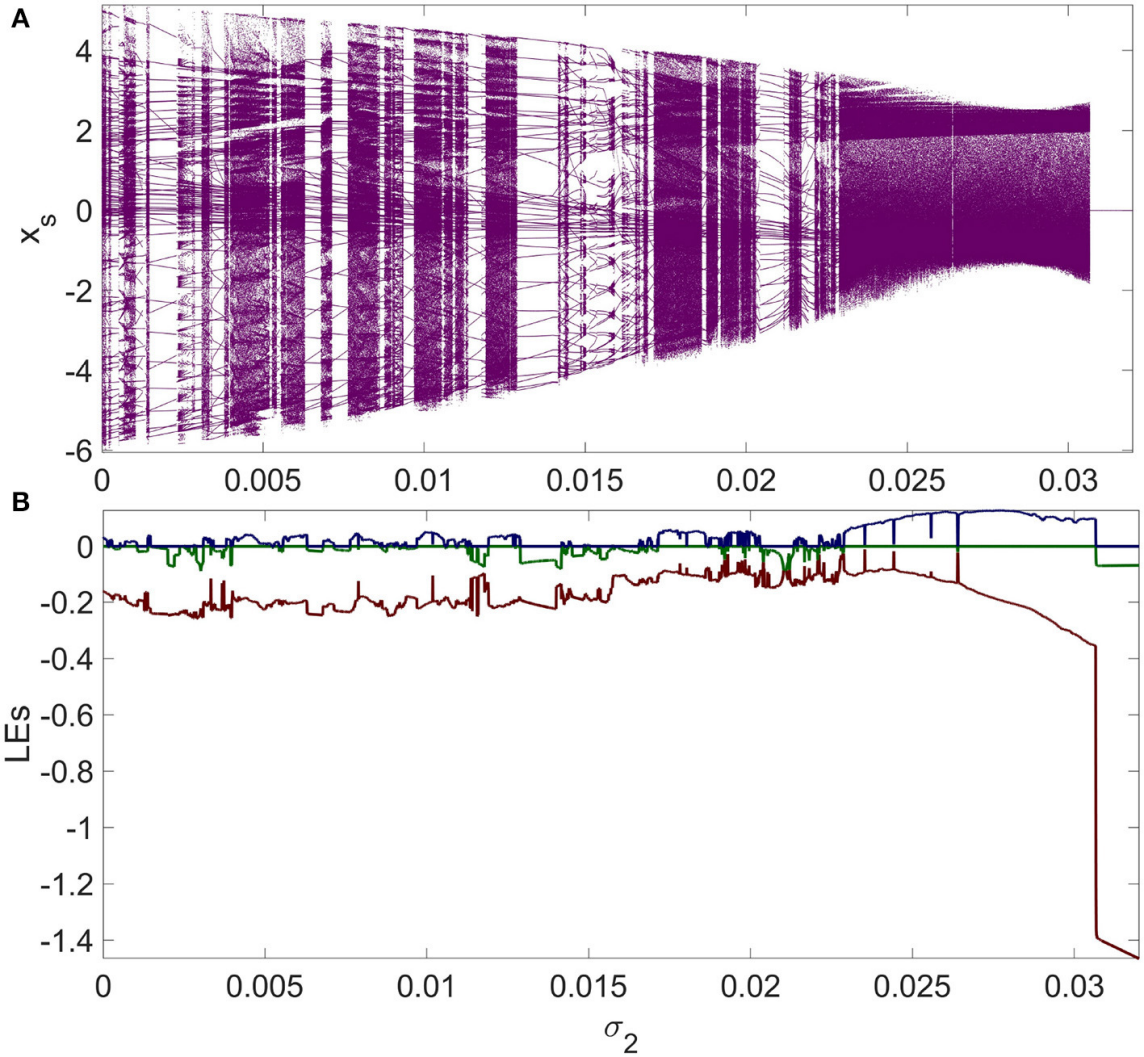
The dynamics of the synchronization manifold defined in System (9) demonstrated as **(A)** the bifurcation diagrams and **(B)** the Lyapunov exponents spectra as a function of the chemical coupling parameter σ_2_, which is, here, the higher-order interactions among mRulkov neurons. Here, *N* = 5 is considered. For specific values of first- and second-order coupling strength found by the MSF analysis and displayed in Figure 4, the mRulkov neurons are capable of achieving synchrony while exhibiting chaotic and periodic behaviors. When in the synchronous state, the dynamics of the neurons are determined by the strength of the higher-order coupling parameter σ_2_.

**Figure 6:**
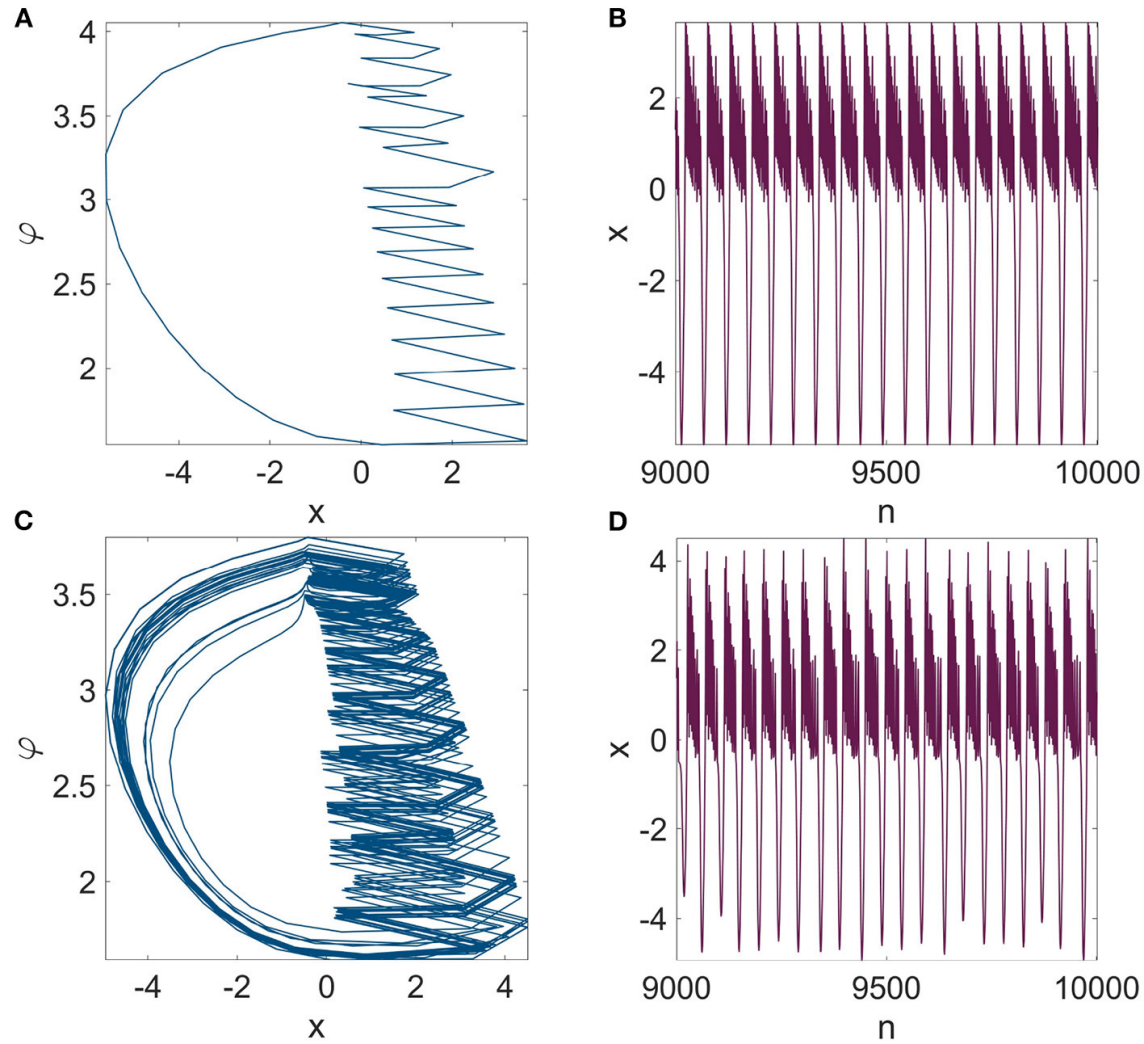
The dynamical behavior of the synchronous mRulkov neurons obtained from Network (5) represented in **(A, C)** *x*-*φ* state space and **(B, D)** time series of variable x over 10,000 iterations. The synchronous mRulkov neurons exhibit periodic behavior for (σ_1_, σ_2_) = (0.1, 0.002) and chaotic dynamics for (σ_1_, σ_2_) = (0.1, 0.01). These two examples demonstrate that the dynamics of the synchronous neurons engaged in Network (5) are distinct from an isolated mRulkov model when subjected to the same parameter settings as those used to obtain Figure 1.

The dynamics of synchronous neurons do not follow the equation for a single isolated neuron, as demonstrated by System (9); nonetheless, they are dependent on the network settings as well as the model parameters, specifically the number of neurons N and the chemical (second-order) coupling parameter σ_2_. Figure 5 illustrates how the dynamics of neurons in the synchronization state, or in other words, the dynamics of System (9), vary according to the coupling parameter σ_2_ by performing a simple dynamical analysis using the bifurcation and the Lyapunov exponents (LEs) diagrams. It can be seen that if first- and second-order coupling parameter values are selected in the synchronous regions demonstrated in Figures 4A, C, the mRulkov neurons are able to exhibit chaotic and periodic behaviors according to the dynamical analysis performed in Figure 5. More precisely, it can be recognized that the neurons synchronize with the dynamics shown in Figure 5 if the first-order coupling strength σ_1_ is chosen in the synchronous zone indicated in Figure 4. For instance, Figures 6A, B shows that *N* = 5 mRulkov neurons in a higher-order network defined in Network (5) achieve synchrony with periodic dynamics (*LE*_1_ = −0.2472, *LE*_2_ = −0.0656, and *LE*_3_ = 0) for σ_1_ = 0.1 and σ_2_ = 0.002; however, if σ_1_ = 0.1 and σ_2_ = 0.01 are selected, as indicated by Figures 6C, D, they behave chaotically (*LE*_1_ = −0.2065, *LE*_2_ = 0, and *LE*_3_ = 0.0499) in the synchronization state.

**Figure 7:**
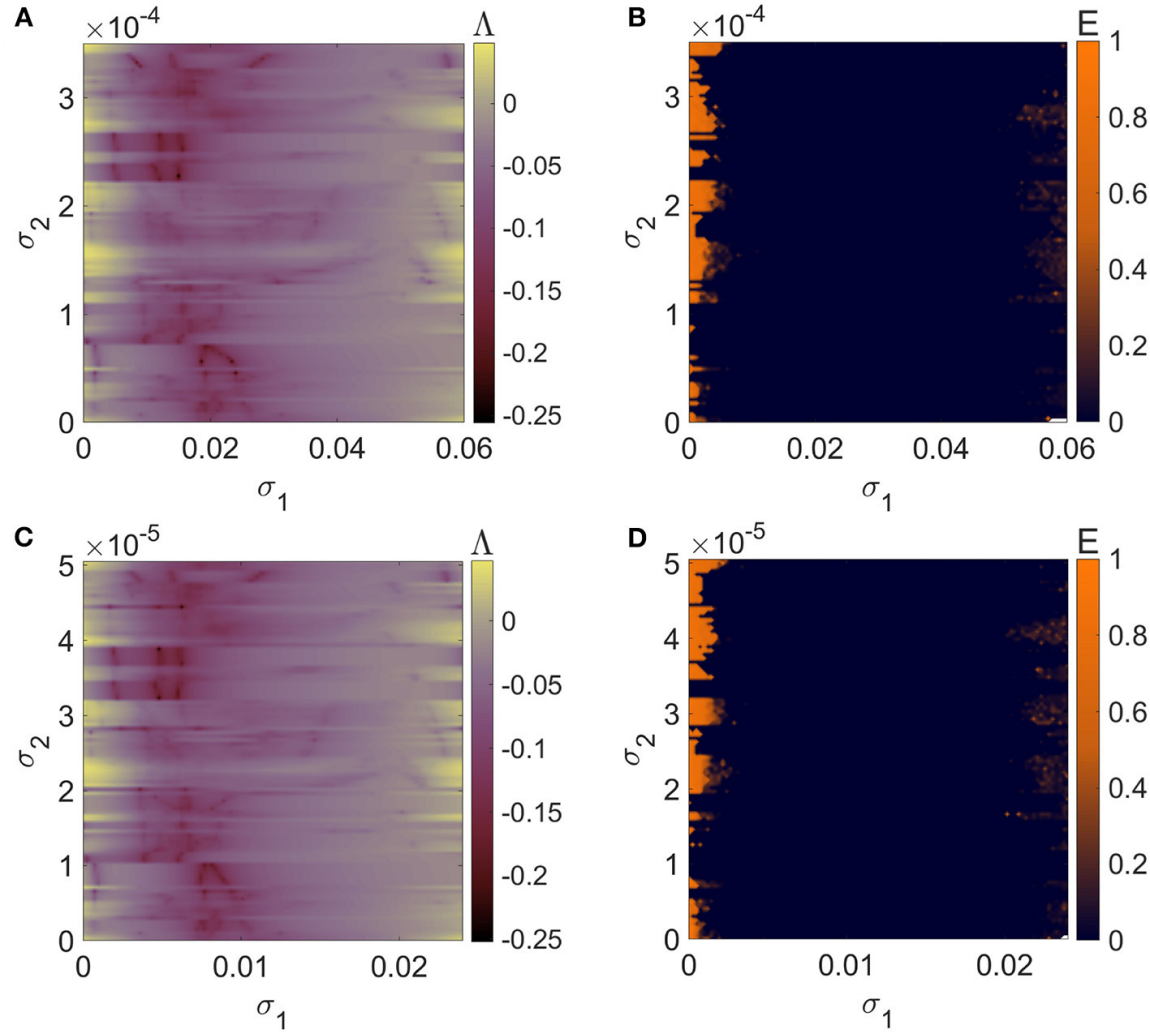
The criteria for synchronizing a network of globally coupled mRulkov neuron maps with pairwise electrical and non-pairwise chemical synapses represented by 2D **(A, C)** maximum Lyapunov exponent of the linearized System (8) and **(B, D)** averaged synchronization error of Network (5). In the first row, *N* = 20, and in the second row, *N* = 50 is considered. When there are more neurons communicating with one another, the synchronization pattern is scaled down to lower values of the coupling parameters. Therefore, as the size of the network increases, synchronization gets enhanced more.

**Figure 8:**
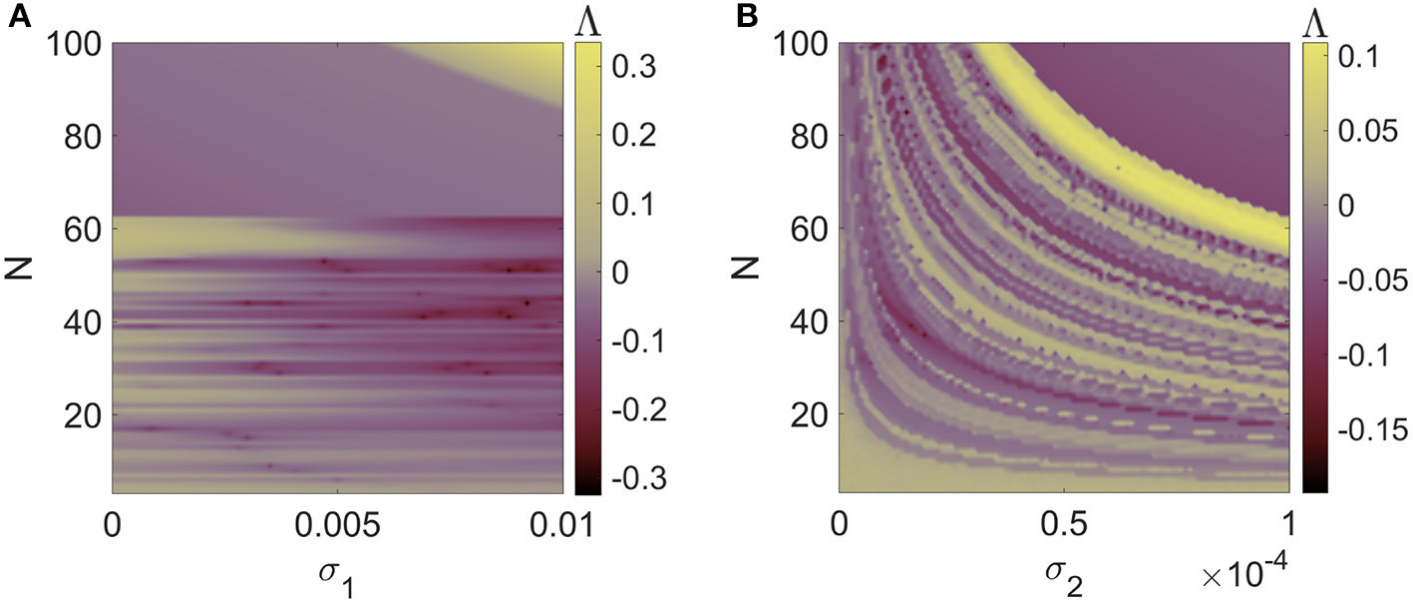
The effect of network size (*N*) on the synchronization of Network (5), including pairwise electrical and non-pairwise chemical interactions, represented in terms of maximum Lyapunov exponent of System (8) as a function of **(A)** σ_1_ for σ_2_ = 0.0001 and **(B)** σ_2_ for σ_1_ = 0.001. An increase in the number of neurons that communicate with one another has a different effect on the synchronization state of the network, depending on whether the first-order (σ_1_) or second-order (σ_2_) coupling strengths are being assessed.

To further examine the impact of network size on the synchronization state of the higher-order network composed of mRulkov models, larger networks with more interacting neurons are taken into consideration. Figures 7A, B demonstrates the synchronous and asynchronous regions of Network (5) for 0 ≤ σ_1_ ≤ 0.06 and 0 ≤ σ_2_ ≤ 0.00035 when *N* = 20 neurons are involved. Similarly, the stability regions of Network (5) with *N* = 50 neurons are 0 ≤ σ_1_ ≤ 0.024 and 0 ≤ σ_2_ ≤ 0.0000505 shown in Figures 7C, D. More precisely, the results show that as the network size increases, the synchronization patterns are scaled to the lower values of both first-order and second-order coupling parameters. However, the amount of this decrease is not the same for σ_1_ and σ_2_. Figure 8 illustrates how the number of participating neurons affects the network's synchronization according to the variation of σ_1_ while σ_2_ = 0.0001 (Figure 8A), and σ_2_ while σ_1_ = 0.001 (Figure 8B).

**Figure 9:**
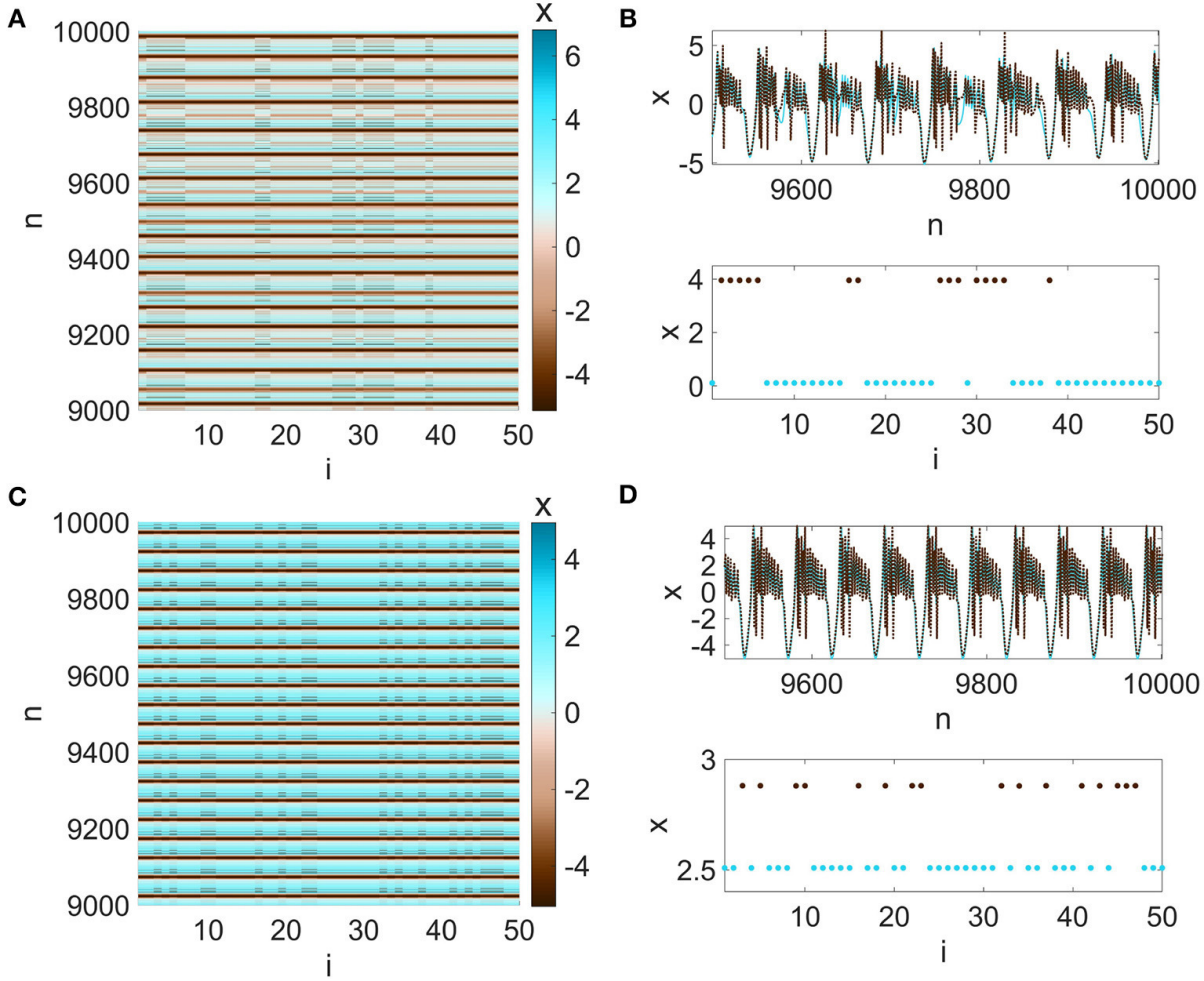
**(A, C)** The spatiotemporal patterns, **(B, D)** neurons' time series (upper panel) and snapshots of the last samples (bottom panel) in Network (5) for (σ_1_, σ_2_) = (0.024, 0.00004) (first row) and (σ_1_, σ_2_) = (0.023, 0.00004) (second row). For stronger strength of electrical (first-order) coupling strength, the neurons tend to form two synchronous clusters with chaotic dynamics (first row) or periodic behavior (second row). In the asynchronous region located on the right side of Figure 4, higher-values of σ_1_ lead to chaotic behaviors, while in lower values, periodic solutions occur in the synchronous clusters.

Focusing on the asynchronous regions in Figures 7C, D, wherein *N* = 50 mRulkov neurons are configured in a higher-order network described by Network (5), cluster synchronization patterns can be detected. More precisely, it is found that the neurons evolve asynchronously in lower values of σ_1_ (in asynchronous regions) while in higher values, they tend to participate in forming synchronous clusters. For instance, as shown in Figures 9A, B, two-cluster synchronization is found for σ_1_ = 0.024 and σ_2_ = 0.00004, in which the synchronous neurons behave chaotically. Based on Figures 9C, D, the same two-cluster synchronization pattern is also identified for σ_1_ = 0.023 and σ_2_ = 0.00004; however, the neurons evolve periodically synchronously in each cluster.

## 6. Conclusions

The consequences of applying higher-order interactions on the synchronization of the mRulkov network were well-explained in this research. In order to achieve this goal, a network of globally connected mRulkov neurons was considered, in which electrical and chemical synapses were respectively applied to the two- and three-node interactions. Thereafter, the regions of the coupling parameter space wherein the neurons were completely synchronous were detected through the MSF approach, which was then verified by calculating the network's synchronization error. The same study was carried out on a network with pure pairwise hybrid interactions, wherein electrical and chemical pathways were considered active simultaneously. Our findings suggest that not neglecting the non-pairwise or multi-node interactions can improve global synchronization by scaling synchronization patterns to lower chemical coupling parameter values while leaving the electrical coupling strength the same. Furthermore, the higher-order coupling strength and network size were demonstrated to alter the behavior of neurons in the synchronization state. Therefore, through the bifurcation analysis, the behaviors of the synchronous neurons, regardless of the stability of the synchronization manifold, were investigated concerning the variation of the second-order chemical coupling parameter. Additionally, it was demonstrated that the neurons could exhibit periodic or chaotic behaviors in the synchronous zone of the parameter space. Through the Lyapunov analysis of the linearized system developed with the MSF formalism, the influence of the engaged neurons' number on the network synchronization was also investigated. The results showed that the synchronous patterns scale to smaller values of the coupling parameters as the network size grows. Furthermore, looking more closely at the asynchronous regions, cluster synchronization patterns were detected. It was shown that the synchronous neurons in the cluster have periodic or chaotic dynamics.

## Data availability statement

The original contributions presented in the study are included in the article/Supplementary material, further inquiries can be directed to the corresponding author.

## Author contributions

MM, SJ, and MP designed and performed the research as well as wrote the paper. All authors contributed to the article and approved the submitted version.
