# Supplementary material for: Synchronization in simplicial complexes of memristive Rulkov neurons
Source: Front Comput Neurosci. 2023 Aug 31;17:1248976. doi: 10.3389/fncom.2023.1248976 (PMC10501309; doi:10.3389/fncom.2023.1248976)
Supplement: Supplementary file 1 [file Presentation_1.zip › Supplementary/Supplementary.pdf]

Appendix: Mathematical Proofs

Letting only hybrid synapses applied describe the interaction between every two neurons (pure pairwise connections), perturbation equations can be expanded as:

$$\begin{aligned}
&\delta \boldsymbol{X}_i(n+1) = J\boldsymbol{F}(\boldsymbol{X}^s)+\\
&\sigma_1 \sum_{j=1}^{N} A_{ij}^{(1)} \times\\
&\Bigg(\frac{\partial \boldsymbol{G}_1^{(1)}(\boldsymbol{X}_i(n), \boldsymbol{X}_j(n))}{\partial \boldsymbol{X}_i(n)}\bigg|_{(\boldsymbol{X}^s(n),\boldsymbol{X}^s(n))} \delta \boldsymbol{X}_i\\
&+\frac{\partial \boldsymbol{G}_1^{(1)}(\boldsymbol{X}_i(n), \boldsymbol{X}_j(n))}{\partial \boldsymbol{X}_j(n)}\bigg|_{(\boldsymbol{X}^s(n),\boldsymbol{X}^s(n))} \delta \boldsymbol{X}_j\Bigg)+\\
&\sigma_2 \sum_{j=1}^{N} A_{ij}^{(1)} \times\\
&\Bigg(\frac{\partial \boldsymbol{G}_2^{(1)}(\boldsymbol{X}_i(n), \boldsymbol{X}_j(n))}{\partial \boldsymbol{X}_i(n)}\bigg|_{(\boldsymbol{X}^s(n),\boldsymbol{X}^s(n))} \delta \boldsymbol{X}_i\\
&+\frac{\partial \boldsymbol{G}_2^{(1)}(\boldsymbol{X}_i(n), \boldsymbol{X}_j(n))}{\partial \boldsymbol{X}_j(n)}\bigg|_{(\boldsymbol{X}^s(n),\boldsymbol{X}^s(n))} \delta \boldsymbol{X}_j\Bigg).
\end{aligned} \tag{A.1}$$

Considering $\boldsymbol{G}_1^{(1)}(\boldsymbol{X}_i(n), \boldsymbol{X}_j(n)) = [x_j(n) - x_i(n), 0, 0]^T$ and $\boldsymbol{G}_2^{(1)}(\boldsymbol{X}_i(n), \boldsymbol{X}_j(n)) = [(v - x_i(n))\Gamma(x_j(n)), 0, 0]$, Eq. (A.1) turns into the following equations:

$$\begin{cases}
\delta x_i(n+1) = Jf(\boldsymbol{X}^s(n))+\\
\sigma_1 \sum_{j=1}^{N} A_{ij}^{(1)}(\delta x_j(n) - \delta x_i(n))+\\
\sigma_2 \sum_{j=1}^{N} A_{ij}^{(1)}((v - x^s(n))\Gamma_x(x^s(n))\delta x_j(n)\\
-\Gamma(x^s(n))\delta x_i(n)),\\
\delta y_i(n+1) = Jg(\boldsymbol{X}^s(n)),\\
\delta \varphi_i(n+1) = Jh(\boldsymbol{X}^s(n)),
\end{cases} \tag{A.2}$$

Assuming $K^{(d)}$ as a tensor whose non-diagonal elements are zero and the diagonal ones are the numbers of d-simplices that the nodes contribute to its construction, the d-order Laplacian matrix can be obtained as $L^{(d)} = K^{(d)} - A^{(d)}$[1]. Accordingly, for the global coupling scheme, the first-order Laplacian matrix can be defined as:

$$L^{(1)} = \begin{cases} -1 & i \neq j \\ N-1 & i = j \end{cases}. \tag{A.3}$$

Therefore, δx_i in Eq. (A.2) can be updated as follows:

$$\begin{aligned}
&\delta x_i(n+1) = Jf(\boldsymbol{X}^s(n))+\\
&\sigma_1 \Bigg(\sum_{j=1}^{N} K_{ij}^{(1)} \delta x_j(n) - \sum_{j=1}^{N} L_{ij}^{(1)} \delta x_j(n)\\
&-\delta x_i(n) \textstyle\sum_{j=1}^{N} A_{ij}^{(1)}\Bigg)+\\
&\sigma_2 \Bigg((v - x^s(n))\Gamma_x(x^s(n))\Bigg(\sum_{j=1}^{N} K_{ij}^{(1)} \delta x_j(n)\\
&-\sum_{j=1}^{N} L_{ij}^{(1)} \delta x_j(n)\Bigg) - \Gamma(x^s(n))\delta x_i(n) \sum_{j=1}^{N} A_{ij}^{(1)}\Bigg)\\
&= Jf(\boldsymbol{X}^s(n)) - \sigma_1 \sum_{j=1}^{N} L_{ij}^{(1)} \delta x_j(n)\\
&+\sigma_2 \Bigg((v - x^s(n))\Gamma_x(x^s(n))\Bigg((N-1)\delta x_i(n) -\\
&\sum_{j=1}^{N} L_{ij}^{(1)} \delta x_j(n)\Bigg) - (N-1)\Gamma(x^s(n))\delta x_i(n)\Bigg).
\end{aligned} \tag{A.4}$$

In Eq. (A.4), $Jf(\boldsymbol{X}^s(n))$ and L are digonalzable. Hence, Eq. (A.4) can be written in terms of the eigenvalues of L, i.e. λ_i, where $\lambda_1 = 0$ and $\lambda_2 = ... = \lambda_N = N$ in global configuration. Finally, the linearized system can be obtained as follows:

$$\begin{cases}
\eta^x(n+1) = Jf(\boldsymbol{X}^s(n)) - \sigma_1 N \eta^x(n) -\\
\sigma_2((v - x^s(n))\Gamma_x(x^s(n))+\\
(N-1)\Gamma(x^s(n)))\eta^x(n),\\
\eta^y(n+1) = Jg(\boldsymbol{X}^s(n)),\\
\eta^\varphi(n+1) = Jh(\boldsymbol{X}^s(n)).
\end{cases} \tag{A.5}$$

Applying chemical synapses to non-pairwise connections while maintaining electrical connections on links, the perturbation equations can be attained through the following:

$$\delta \boldsymbol{X}_i(n+1) = J\boldsymbol{F}\big(\boldsymbol{X}^s\big)+ \\ \sigma_1 \sum_{j=1}^{N} A_{ij}^{(1)} \times \\ \Big(\frac{\partial \boldsymbol{G}^{(1)}\big(\boldsymbol{X}_i(n),\boldsymbol{X}_j(n)\big)}{\partial \boldsymbol{X}_i(n)}\Big|_{\big(\boldsymbol{X}^s(n),\boldsymbol{X}^s(n)\big)} \delta\boldsymbol{X}_i \\ +\frac{\partial \boldsymbol{G}^{(1)}\big(\boldsymbol{X}_i(n),\boldsymbol{X}_j(n)\big)}{\partial \boldsymbol{X}_j(n)}\Big|_{\big(\boldsymbol{X}^s(n),\boldsymbol{X}^s(n)\big)} \delta\boldsymbol{X}_j\Big)+ \\ \sigma_2 \sum_{j=1}^{N}\sum_{k=1}^{N} A_{ijk}^{(2)} \times \\ \Big(\frac{\partial \boldsymbol{G}^{(2)}\big(\boldsymbol{X}_i(n),\boldsymbol{X}_j(n),\boldsymbol{X}_k(n)\big)}{\partial \boldsymbol{X}_i(n)}\Big|_{\big(\boldsymbol{X}^s(n),\boldsymbol{X}^s(n),\boldsymbol{X}^s(n)\big)} \delta\boldsymbol{X}_i \\ +\frac{\partial \boldsymbol{G}^{(2)}\big(\boldsymbol{X}_i(n),\boldsymbol{X}_j(n),\boldsymbol{X}_k(n)\big)}{\partial \boldsymbol{X}_j(n)}\Big|_{\big(\boldsymbol{X}^s(n),\boldsymbol{X}^s(n),\boldsymbol{X}^s(n)\big)} \delta\boldsymbol{X}_j \\ +\frac{\partial \boldsymbol{G}^{(2)}\big(\boldsymbol{X}_i(n),\boldsymbol{X}_j(n),\boldsymbol{X}_k(n)\big)}{\partial \boldsymbol{X}_k(n)}\Big|_{\big(\boldsymbol{X}^s(n),\boldsymbol{X}^s(n),\boldsymbol{X}^s(n)\big)} \delta\boldsymbol{X}_k\Big). \tag{A.6}$$

Letting $\boldsymbol{G}^{(1)}\big(\boldsymbol{X}_i(n),\boldsymbol{X}_j(n)\big) = [x_j(n)-x_i(n),0,0]^T$ and $\boldsymbol{G}^{(2)}\big(\boldsymbol{X}_i(n),\boldsymbol{X}_j(n),\boldsymbol{X}_k(n)\big) = \big[(v - x_i(n))\big(\Gamma(x_j(n))\Gamma(x_k(n))\big),0,0\big]$ as the first-order and second-order interactions, Eq. (A.6) can be clarified as follows:

$$\begin{cases} \delta x_i(n+1) = Jf\big(\boldsymbol{X}^s(n)\big)+ \\ \sigma_1 \sum_{j=1}^{N} A_{ij}^{(1)}\big(\delta x_j(n)-\delta x_i(n)\big)+ \\ \sigma_2 \sum_{j=1}^{N}\sum_{k=1}^{N} A_{ijk}^{(2)}\big((v-x^s(n)) \\ \big(\Gamma_x(x^s(n))\Gamma(x^s(n))\big)(\delta x_j(n)+\delta x_k(n))\times \\ -\Gamma^2(x^s(n))\delta x_i(n)\big), \\ \delta y_i(n+1) = Jg\big(\boldsymbol{X}^s(n)\big), \\ \delta\varphi_i(n+1) = Jh\big(\boldsymbol{X}^s(n)\big), \end{cases} \tag{A.7}$$

Deeming $L^{(d)} = K^{(d)} - A^{(d)}$ as a general relation and global configuration, the second-order Laplacian matrix is defined as:

$$L^{(2)} = \begin{cases} 0 & i \neq j \, and \, A_{ij}^{(1)} = 0 \\ -(N-2) & i \neq j \, and \, A_{ij}^{(1)} = 1 \\ (N-1)(N-2) & i = j \end{cases}. \tag{A.8}$$

As a result, δx_i in Eq. (A.7) can be rewritten as follows:

$$\delta x_i(n+1) = Jf\big(\boldsymbol{X}^s(n)\big)+ \\ \sigma_1\Big(\sum_{j=1}^{N} K_{ij}^{(1)}\delta x_j(n) - \sum_{j=1}^{N} L_{ij}^{(1)}\delta x_j(n) \\ -\delta x_i(n)\textstyle\sum_{j=1}^{N} A_{ij}^{(1)}\Big)+ \\ \sigma_2\Big(\big(v-x^s(n)\big)\big(\Gamma_x(x^s(n))\Gamma_x(x^s(n))\big)\times \\ \Big(\sum_{j=1}^{N}\sum_{k=1}^{N} K_{ijk}^{(2)}(\delta x_j(n)+\delta x_k(n)) \\ -\sum_{j=1}^{N}\sum_{k=1}^{N} L_{ijk}^{(2)}(\delta x_j(n)+\delta x_k(n))\Big)- \\ \Gamma^2(x^s(n))\delta x_i(n)\sum_{j=1}^{N}\sum_{k=1}^{N} A_{ijk}^{(2)}\Big) \\ = Jf\big(\boldsymbol{X}^s(n)\big) - \sigma_1\sum_{j=1}^{N} L_{ij}^{(1)}\delta x_j(n) \\ +\sigma_2\Big(\big(v-x^s(n)\big)\big(\Gamma_x(x^s(n)\Gamma(x^s(n))\big)\times \\ \Big(2(N-1)(N-2)\delta x_i(n)- \\ \sum_{j=1}^{N}\sum_{k=1}^{N} L_{ijk}^{(2)}(\delta x_j(n)+\delta x_k(n))\Big)- \\ (N-1)(N-2)\Gamma^2(x^s(n))\delta x_i(n)\Big). \tag{A.9}$$

Since $\sum_{j=1}^{N} L_{ij}^{(2)}\delta x_j(n) = \sum_{k=1}^{N} L_{ik}^{(2)}\delta x_k(n)$ and $L^{(2)} = (N-2)L^{(1)}$ for a global coupling scheme[2], Thus:

$$\delta x_i(n+1) = Jf\big(\boldsymbol{X}^s(n)\big) - \sigma_1\sum_{j=1}^{N} L_{ij}^{(1)}\delta x_j(n) \\ +\sigma_2(N-2)\Big(2(v-x^s(n))\big(\Gamma_x(x^s(n)\Gamma(x^s(n))\big)\times \\ \Big((N-2)\delta x_i(n) - \sum_{j=1}^{N} L_{ij}^{(1)}\delta x_j(n)\Big)- \\ (N-1)\Gamma^2(x^s(n))\delta x_i(n)\Big), \tag{A.10}$$

and similar to the previous case, the simplified perturbation system can be projected to the following linearized system:

$$\begin{cases} \eta^x(n+1) = Jf\big(\boldsymbol{X}^s(n)\big) - \sigma_1 N\eta^x(n)- \\ \sigma_2(N-2)\big(2(v-x^s(n))\times \\ \big(\Gamma_x(x^s(n)\Gamma(x^s(n))\big)+ \\ (N-1)\Gamma^2(x^s(n))\big)\eta^x(n), \\ \eta^y(n+1) = Jg\big(\boldsymbol{X}^s(n)\big), \\ \eta^\varphi(n+1) = Jh\big(\boldsymbol{X}^s(n)\big), \end{cases} \tag{A.11}$$

REFERENCES

[1] L. V. Gambuzza, F. Di Patti, L. Gallo, S. Lepri, M. Romance, R. Criado, M. Frasca, V. Latora, and S. Boccaletti, "Stability of synchronization in simplicial complexes," Nat. Commun. **12**, 1255 (2021).

[2] M. Lucas, G. Cencetti, and F. Battiston, "Multiorder Laplacian for synchronization in higher-order networks," Phys. Rev. Res. **2**, 033410 (2020).
